# Digital Inspection Technology for Sheet Metal Parts Using 3D Point Clouds

**DOI:** 10.3390/s25154827

**Published:** 2025-08-06

**Authors:** Jian Guo, Dingzhong Tan, Shizhe Guo, Zheng Chen, Rang Liu

**Affiliations:** College of Mechanical and Electrical Engineering, Harbin Engineering University, Harbin 150001, China; guojian_2006@hrbeu.edu.cn (J.G.); 19845181336@163.com (S.G.); chenzheng8895@163.com (Z.C.); liurang122@163.com (R.L.)

**Keywords:** digital measurement, point cloud data, point cloud registration, three-dimensional reconstruction

## Abstract

To solve the low efficiency of traditional sheet metal measurement, this paper proposes a digital inspection method for sheet metal parts based on 3D point clouds. The 3D point cloud data of sheet metal parts are collected using a 3D laser scanner, and the topological relationship is established by using a K-dimensional tree (KD tree). The pass-through filtering method is adopted to denoise the point cloud data. To preserve the fine features of the parts, an improved voxel grid method is proposed for the downsampling of the point cloud data. Feature points are extracted via the intrinsic shape signatures (ISS) algorithm and described using the fast point feature histograms (FPFH) algorithm. After rough registration with the sample consensus initial alignment (SAC-IA) algorithm, an initial position is provided for fine registration. The improved iterative closest point (ICP) algorithm, used for fine registration, can enhance the registration accuracy and efficiency. The greedy projection triangulation algorithm optimized by moving least squares (MLS) smoothing ensures surface smoothness and geometric accuracy. The reconstructed 3D model is projected onto a 2D plane, and the actual dimensions of the parts are calculated based on the pixel values of the sheet metal parts and the conversion scale. Experimental results show that the measurement error of this inspection system for three sheet metal workpieces ranges from 0.1416 mm to 0.2684 mm, meeting the accuracy requirement of ±0.3 mm. This method provides a reliable digital inspection solution for sheet metal parts.

## 1. Introduction

At present, the measurement of sheet metal parts is mainly divided into two kinds: contact measurement and non-contact measurement [1,2]. Contact measurement has strong versatility, but it has some drawbacks. For example, the measurement speed is slow, and it requires contact with the surface of the workpiece. As a result, the workpiece is prone to deformation, and scratches may occur on the surface. At the same time, this method has relatively high technical requirements for workers. Non-contact measurement refers to the measurement of the workpiece through the principles of optics, electromagnetism, acoustics, etc., without contact with the surface of the workpiece, thus reducing the additional loss of the workpiece. The most common methods of non-contact measurement are monocular measurement, binocular measurement, structured light measurement and laser measurement.

Laser measurement technology, as an advanced detection means, has been widely applied in aerospace and other fields. Schalk et al. [3] designed a pipe measuring system based on laser triangulation to measure the size of rolled seamless pipes. The pipe size is obtained by fitting the circle and plane with the least squares method. Krzysztof Magda et al. [4] designed a measurement system to obtain the surface profiles of logs, which consists of six laser scanners that can scan logs from 250 mm to 500 mm in diameter and up to 4000 mm in length. Tong et al. [5] designed a contactless measuring system for the measurement of thread parameters. After obtaining the contour data points, the measurement system fits and partitions the data and calculates parameters such as the thread pitch and pitch diameter based on the processed data. Cai et al. [6] designed a test system for the measurement, reconstruction and calculation of the volumes of potatoes. In this system, the potato is scanned by a line laser scanner, the lost top and bottom point clouds are repaired via interpolation methods and three-dimensional reconstruction is carried out. Finally, the linear regression method is used to determine the linear relationship between the measured potato volume and the actual potato volume and calculate the density of the potato.

The point cloud data obtained from workpiece scanning by a 3D laser scanner should be denoised, registered and 3D-reconstructed. Haque et al. [7] proposed a method for the robust noise reduction of point clouds while retaining fine features. Outliers of point clouds are detected and removed utilizing a dissimilarity measure based on point positions and their corresponding normals, and the bilateral filtering method is used for the noise reduction of point clouds. Lee et al. [8] proposed an algorithm based on the voxel grid method for data preprocessing. This method removes the noise in the point cloud, reduces the point cloud data by about 40% and removes 13% of the overlapping points to reduce the number of point clouds. Heinzler et al. [9] proposed a method based on convolutional neural networks to denoise point cloud data, which can significantly improve the denoising effect in light detection and ranging (LiDAR) point cloud data under poor weather conditions.

Ren et al. [10] proposed a multi-scale noise filtering algorithm. Firstly, the algorithm calculates the normal vectors of the point cloud data and the three eigenvalues of the sampling points through principal component analysis, and it distinguishes noise of different scales according to the relationship between the variation factor of the sampling point surface and the average surface factor. Then, the large-scale noise of the flat region and the small-scale noise of the abrupt region are filtered via a statistical filtering algorithm and bilateral filtering algorithm, respectively. The processed data can better display the surface of the rock. Bochang Zou et al. [11] proposed an improved method to address the problem whereby using a centroid to replace all point clouds within a certain range during the downsampling process leads to the loss of some feature points of the point clouds within this range. This method uses downsampling to find the centroid, adds it to the farthest point sampling and conducts ten iterations. The distances of the obtained 11 points are weighted and averaged to find the feature points. This algorithm simplifies 90,840 points within 48 s, while retaining the fine features of the point clouds. Zhao et al. [12] proposed a point cloud preprocessing method for the detection of workpiece surface defects. This method uses a laser scanner to obtain the 3D point cloud data of the workpiece and adopts the K-nearest neighbors (KNN) algorithm to reduce the data density. While retaining the data characteristics, it compresses the point cloud data to 60.20% of the original data.

Makovetskii et al. [13] proposed an improved algorithm based on the point-to-point ICP registration algorithm. This method uses points and normal vectors to align 3D point clouds, while the common point-to-point approach uses only the coordinates of points. At the same time, a regularization term is added to find a suitable transformation matrix between point clouds with poor correspondence, which helps to improve the accuracy of point cloud registration. Koide et al. [14] proposed a voxelized generalized iterative closest point algorithm. By combining the generalized iterative closest point (GICP) method with voxelization, it reduces the time required for the nearest neighbor search. Compared with the GICP and normal distributions transform (NDT) algorithms, this algorithm has better registration accuracy and efficiency. Young et al. [15] proposed a grid-based GICP algorithm for point cloud registration. Compared with the traditional GICP algorithm, the registration range of this method is 4 to 17 times larger. At the same time, it is more accurate and faster than the traditional GICP algorithm in registering sparse or non-uniform point clouds.

Yu et al. [16], addressing problems such as long calculation times and poor registration accuracy in point cloud registration, proposed an improved ICP algorithm based on matching points. This method first uses the RANSAC algorithm to segment the point cloud data obtained by LiDAR and filters out abnormal matching points. Finally, it combines the KD tree with ICP for point cloud registration. This method combines the advantages of point cloud filtering and the secondary filtering of matching point pairs, improving the calculation speed and registration accuracy. He et al. [17] proposed a new registration algorithm that combines PointNet++ and ICP. It samples and extracts the local feature descriptors of the input point cloud. Then, according to the feature descriptors, it finds the corresponding points required for ICP matching in the source point cloud and the target point cloud to estimate the rotation and translation matrices, reducing the calculation time required and decreasing the dependence on the initial values.

Vizzo et al. [18] proposed an improved algorithm based on Poisson surface reconstruction to obtain the specific shape of terrain. The point cloud data obtained through multiple scans are registered to obtain the overall point cloud data of the terrain, and then 3D reconstruction is carried out, resulting in a relatively accurate map. Maurya et al. [19] proposed a reconstruction method based on the greedy triangulation algorithm to reconstruct the point cloud data of a coastal dune area obtained by a 3D scanner. They used KD tree search, local meshing and surface angle and search radius limitations to process the point clouds in the coastal dune area, achieving the reconstruction of coastal dunes. The reconstruction integrity was between 93.4% and 96.8%. Ando et al. [20] proposed a 3D reconstruction algorithm to reconstruct the surfaces of plant leaves. They achieved the 3D reconstruction of the leaves of two crops: soybeans and beets. Compared with the traditional Poisson reconstruction algorithm, this algorithm can reduce the influence of noise and better reconstruct the leaf surface and has good robustness.

Gu et al. [21] implemented the preprocessing of point cloud data based on the C++ point cloud library, including filtering and smoothing, feature extraction and hole filling. The greedy projection triangulation method and the Poisson reconstruction method were applied to conduct the 3D reconstruction of the point cloud data. The error of the circle reconstructed by Poisson reconstruction was 0.195%, and the error of the circle reconstructed by greedy triangulation was 0.178%. In order to reconstruct a rose fruit model with complex geometric features and high precision, Xie et al. [22] proposed a 3D point cloud reconstruction method for Rosa roxburghii fruits based on a 3D laser scanner, which included 3D point cloud acquisition, point cloud registration, fruit segmentation and 3D reconstruction. Compared with the volume measured manually, the error in the volume of the reconstructed fruit was 1.01%. Pan et al. [23] proposed a segmentation method based on voxel structures and global optimization that can effectively separate bridge features. At the same time, in [23], the authors reconstructed the Hongde Bridge and the Tongxin Bridge. The reconstruction accuracies were 83% and 80%, respectively, and they were able to better reconstruct features such as the bridge deck and the fence.

In summary, scholars from various countries have achieved certain results in point cloud preprocessing technology. Some algorithms simplify the point cloud data after downsampling but fail to completely retain the features of the point clouds, resulting in poor results in subsequent operations on the point cloud data. Point cloud registration technology usually includes rough registration and fine registration. The accuracy of rough registration is generally low, while the accuracy of fine registration is high but it takes a long time. At present, the effects of 3D point cloud registration in terms of registration accuracy and speed are still not ideal. When dealing with large point cloud data and cases in which the initial position of the point cloud is not satisfactory, both the registration rate and the registration accuracy are poor. The models obtained after current 3D reconstruction have holes, and there is a large difference from the actual objects, making it impossible to accurately retain the features of these objects.

This paper takes aircraft sheet metal components as the research objects. Based on the digital detection technology for sheet metal parts using 3D point clouds, a digital detection system based on a laser scanner is constructed. The workpiece is scanned by a 3D laser scanner to obtain point cloud data. The geometric dimensions of the workpiece are measured based on methods such as point cloud data processing and planarization. By comparing the measured dimensions of the workpiece with the actual dimensions, it is shown that the proposed digital detection method can meet the measurement accuracy requirements.

The rest of the paper is organized as follows. Section 2 describes the digital detection method for sheet metal parts based on 3D point clouds, including the acquisition and preprocessing of point cloud data, point cloud registration, 3D reconstruction and other aspects. In Section 3, the experiments on point cloud denoising, registration and the 3D reconstruction of sheet metal parts are presented. Section 4 presents the conclusions of this paper.

## 2. Digital Inspection Method for Sheet Metal Parts Using 3D Point Clouds

### 2.1. Acquisition and Preprocessing of Point Cloud Data of Sheet Metal Parts

In this paper, the EinScan Pro 2X 3D scanner (Shining 3D Technology Co., Ltd., Hangzhou, China) is used to scan sheet metal parts to obtain point cloud files in ply format. The ply format files are then converted into pcd format files, which enable the processing of the point cloud data using the PCL library. The specific parameters of the EinScan Pro 2X are shown in Table 1. When using this scanner to measure the dimensions of sheet metal parts, it can meet the requirement for measurement accuracy of ±0.3 mm.

(1) Establishment of point cloud topological relationship of sheet metal parts

In this paper, the KD tree method is adopted to construct the topological structure. The parameter of the KD tree is set to 20—that is, 20 points are used to construct the topological structure. This value is determined based on the performance of the scanning equipment and the dimensions of the measured part used in this paper. The steps to construct a 3D KD tree are as follows. First, determine the splitting plane. Calculate the variances of the 3D data in the x, y, and z directions and select the coordinate axis with the largest variance as the splitting axis. Then, sort the data on the selected coordinate axis and use the median point as the splitting point to establish a splitting plane that passes through this point and is perpendicular to the selected axis. In this way, the data points are divided into two parts: those with coordinate values less than the median point form the left subtree, and those with coordinate values greater than the median point form the right subtree. By recursively repeating this process, continue to construct the left and right subtrees until there are no more data points that can be split, and the construction of the 3D KD tree is completed.

When using the KD tree method to establish the topological relationship of point clouds and dealing with large-scale point cloud data, constructing a KD tree may be time-consuming and require a large amount of memory resources.

(2) Point cloud filtering and processing of sheet metal parts

The preprocessing of point cloud data, such as denoising and downsampling, can reduce the amount of data and noise, thus optimizing the processing time and improving the processing accuracy.

① Pass-Through Filtering Algorithm

The principle of the pass-through filtering algorithm is to determine whether to retain a point based on the coordinate values of the points in the point cloud data. Its main principle is to select one of the coordinate axes—namely the x-axis, y-axis, or z-axis—for filtering. Then, a filtering range [a, b] is specified. All point cloud data are traversed, and only the points whose coordinate values on the selected axis fall within the filtering range are retained, while the other points are filtered out.

**Figure 1:**
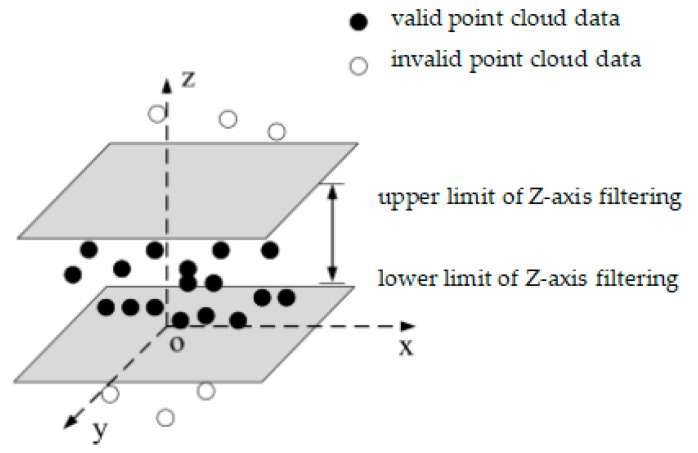
Schematic diagram of the pass-through filtering algorithm.

This paper uses the pass-through filtering algorithm to filter the point cloud data. The interval of the x-axis is [20.7, 100.5], the interval of the y-axis is [40.6, 120.4], and the interval of the z-axis is [6.8, 11.6]. These values are determined based on the size of the measured part, aiming to completely retain the point clouds that represent the features of the part. All point cloud data within these intervals are retained, while the other point cloud data are deleted to complete the pass-through filtering. A schematic diagram of the pass-through filtering algorithm is shown in Figure 1.

② Downsampling and Processing for Improved Voxel Grid Filtering

For the collected point cloud data, the voxel grid filtering algorithm is adopted to achieve their simplification [24]. When applying the voxel grid method, the calculated centroid points of each voxel grid may not be the actual coordinate points in the original point cloud data. Directly using these centroid points to represent the entire voxel grid may not allow the fine features of the original point cloud to be retained. Therefore, in this paper, the nearest neighbor search method is used to find the point in the original point cloud that is closest to the centroid within each voxel grid, and this point is used to represent all points of the entire voxel. In this way, it is ensured that the downsampled point cloud data still originate from the original data set, and, at the same time, the fine features of the original point cloud can be retained. The specific steps are as follows:

(1) Suppose that the original point cloud data set P contains N data coordinate points, and the voxel grid method is adopted to perform downsampling on the point cloud data.
(2) Calculate the centroid coordinate points, *p*(*x*, *y*, *z*), of each non-empty voxel grid and construct a new set of centroid points, *p_i_*(*x_i_, y_i_, z_i_*).
(3) Search each voxel grid according to the KD tree nearest neighbor search method and take the point closest to the centroid in it as the new downsampled point to obtain a new set of centroid points, *p_i_*.

In this paper, an improved voxel grid filtering algorithm is used for downsampling. The size of the voxel grid is set to [1.3, 1.3, 1.3]. These values are determined based on the size of the measured part and the number of point clouds obtained per unit area by the scanning equipment, aiming to reduce the number of retained point clouds while preserving the image features of the point cloud data, as well as reducing the amount of computation. The KD tree is used to search for the point closest to the centroid to replace the centroid in order to complete the simplification of the point cloud data.

### 2.2. Point Cloud Registration of Sheet Metal Parts

#### 2.2.1. Rough Registration of Point Clouds

(1) ISS feature point extraction algorithm

The ISS algorithm is a point cloud feature detection algorithm used to extract feature points from point cloud data. It is applied to identify and describe surface features. It can be applied in high-quality initial point cloud registration [25]. Suppose that the point cloud data P contain N data points, and the coordinate of any point *p_i_* is *p_i_*(*x_i_, y_i_, z_i_*), *i* = 0, 1, 2,…, *N* − 1. The specific algorithm process is as follows.

① A local coordinate system is established for each point *p_i_* in the point cloud data, and a search radius *r* is set for all points. An appropriate search radius *r* needs to be selected based on the actual situation, such as the density of the point cloud.
② Query all points in the area of radius *r* centered on each point *p_i_* in point cloud data P and calculate the weights *w_ij_* of these points, as shown in Equation (1):
  (1) $$w_{ij}=\frac{1}{\left|p_{i}-p_{j}\right|},|p_{i}-p_{j}|<r$$
③ Calculate the covariance matrix of each point *p_i_*, as shown in Equation (2):
  (2) $$\mathrm{cov}(p_{i})=\frac{\sum_{|p_{i}-p_{i}|<r}w_{ij}(p_{i}-p_{j}){\left(p_{i}-p_{j}\right)}^{T}}{\sum_{|p_{i}-p_{i}|<r}w_{ij}}$$
④ Calculate the eigenvalues $\left\{\lambda_{i}^{1},\lambda_{i}^{2},\lambda_{i}^{3}\right\}$ of covariance matrix cov(*p_i_*) for each point *p_i_* in the point cloud data and arrange them in order from largest to smallest.
⑤ Set the threshold values $\varepsilon_{1}$ and $\varepsilon_{2}$, and the points that meet the screening conditions of Equation (3) are ISS feature points.
  (3) $$\frac{\lambda_{i}^{2}}{\lambda_{i}^{1}}\leq \varepsilon_{1},\frac{\lambda_{i}^{3}}{\lambda_{i}^{1}}\leq \varepsilon_{2}$$

$\varepsilon_{1}$ is used to judge whether a point is a marginal point. The larger this value is, the more likely it is that the point is an edge point. $\varepsilon_{2}$ is used to determine whether a point is a corner point; the larger the value is, the more likely it is that the point is a corner point.

**Figure 2:**
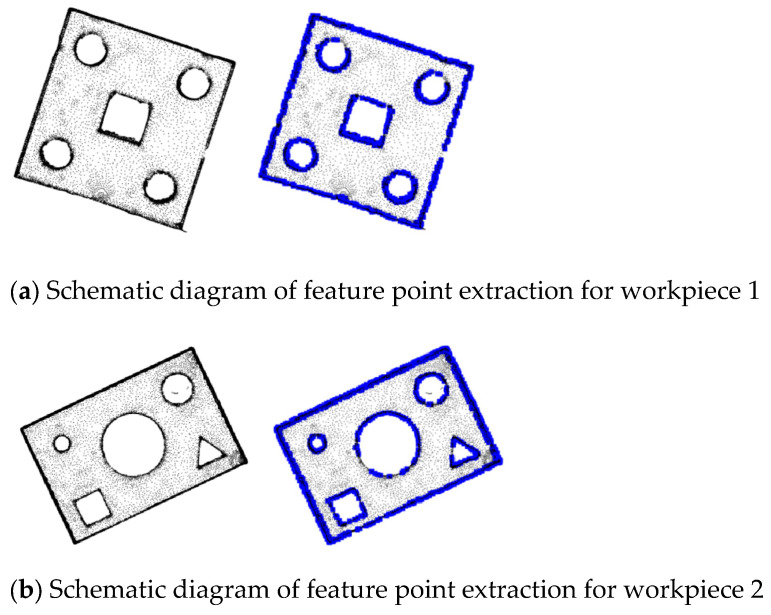
ISS feature point extraction diagrams of different sheet metal parts.

The feature points of two workpieces are extracted through the ISS feature point extraction algorithm, and the experimental results are shown in Figure 2.

Through the ISS algorithm used to extract features of different sheet metal parts, it can be seen that this algorithm deletes most of the points located on the plane of the sheet metal part and retains the blue key points, including edge points, corner points, etc., which can reflect the shape of the sheet metal part well.

(2) FPFH feature description

FPFH is an improvement method based on point feature histograms (PFH). They reduce the computational complexity and the amount of calculation. FPFH introduces the method of histogram statistics for the attributes of adjacent points, avoiding the complex feature calculation process of PFH. It retains, as much as possible, the description of the local geometric information of the point cloud, and the calculation speed is fast.

The calculation steps of FPFH are as follows.

① For the point *P_q_* to be calculated, take it as the center, with r as the radius, and find the nearest *k* neighboring points in the neighborhood space. Then, connect the remaining points in the neighborhood space with the points *P_q_* and find the corresponding four values (α,ϕ,θ,d) so as to express the position relationship between the two points. Thus, the simplified point feature histogram SPFH is established and denoted as S(Mq).

② For all neighboring points $P_{k}$ of the point *P_q_*, re-establish the neighborhood space with *r* as the radius according to step ①, search *k* neighboring points $P_{k}$ in the neighborhood space and obtain the SPFH value of each nearby point $P_{k}$, denoted as $S(M_{q})$. According to this value, calculate the FPFH value of point *P_q_*, denoted as $S(M_{q})$. The calculation formula is as follows:

(4) $$F(M_{q})=S(M_{q})+\frac{1}{k}\sum_{i=1}^{k}\frac{1}{w_{k}}S(M_{k})$$

where $w_{k}$ is the weighted value of the *k*-th adjacent point of point *P_q_*; $\frac{1}{w_{k}}$ is the distance between the calculated point *P_q_* and the *k*-th neighboring point.

**Figure 3:**
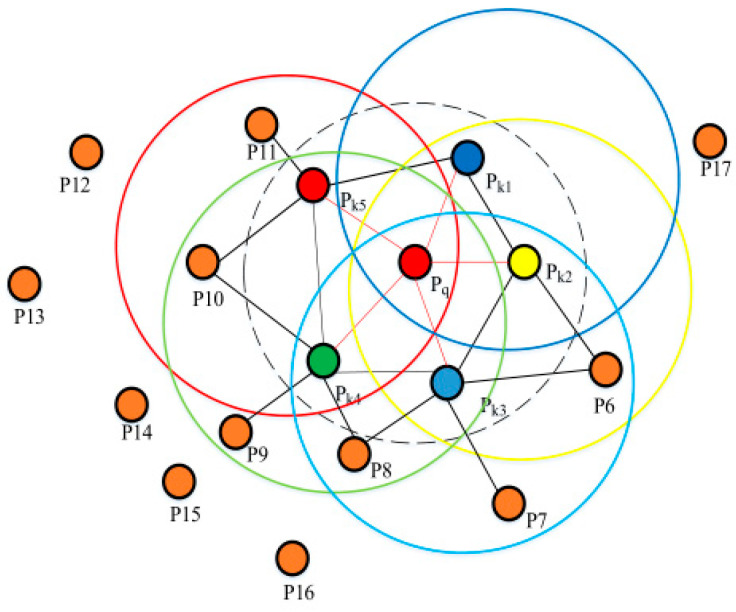
FPFH of point *P_q_*.

The FPFH of point *P_q_* is shown in Figure 3.

**Figure 4:**
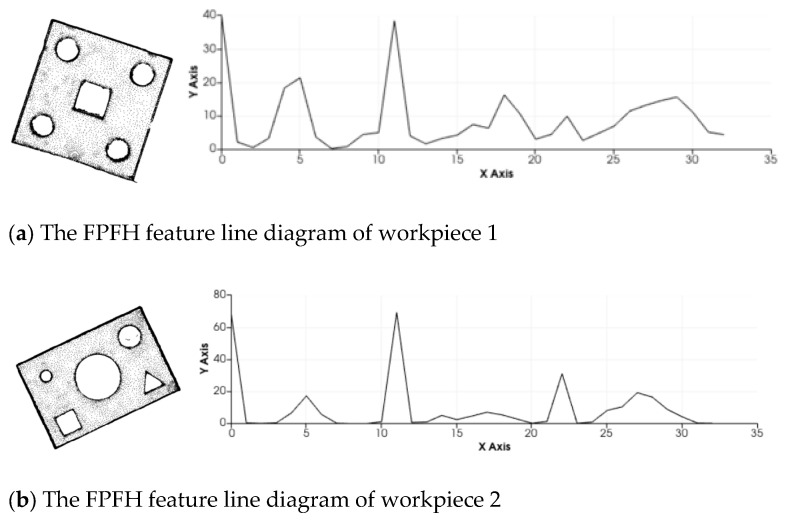
The FPFH feature line diagrams of different sheet metal parts.

The FPFH descriptor is used to describe the features of the two sheet metal parts, as shown in Figure 4.

In the FPFH feature histogram in Figure 4, the range [0, 35] on the x-axis represents the relevant components of the surface shape of the sheet metal part, and the y-axis represents the FPFH values in different intervals. It can be seen that the FPFH line charts of the different sheet metal parts have significant differences.

(3) SAC-IA algorithm

The ISS algorithm is used to extract point cloud feature points, the FPFH algorithm is used to describe point cloud features, and the SAC-IA algorithm is used for rough registration. The specific steps of the SAC-IA algorithm are as follows.

① Selection of sampling points: Select points to be matched from the source point cloud to be registered and set a distance threshold *d*. Ensure that the distance between the points to be matched is greater than *d* to avoid the repeated or overly concentrated selection of point clouds. Guarantee that the selected points to be matched are relatively dispersed, and ensure that the FPFH values of the sampling points are different.

② Search for corresponding points regarding the sampling points: According to the calculated FPFH features of the point cloud to be matched, search for points with the same or similar features to the sampling points from the target point cloud and take them as the corresponding points to the points to be matched in the source point cloud to be registered.

③ Remove the incorrect matching points: Set the corresponding distance *l* of the matching points, which is used to remove incorrect corresponding points in order to improve the accuracy of registration.

④ Set the number of iterations: In order to prevent the algorithm from failing to converge and to limit the running time of the algorithm, a maximum number of iterations is set [26].

⑤ Calculate the transformation matrix: Calculate the rotation matrix and the translation matrix for all sets of corresponding point pairs. Then, calculate the “sum of distance errors” function between the corresponding point sets according to the calculated transformation matrix. This function is used as an indicator for the evaluation of the registration performance. See Equation (5):

(5) $$H(l_{i})=\left\{\begin{array}{c} \frac{1}{2}l_{i}^{2},||l_{i}||<m_{l}\\ \frac{1}{2}m_{l}(2||l_{i}-m_{l}),||l_{i}||>m_{l} \end{array}\right.$$

where $m_{1}$ is a fixed value set in advance; $l_{i}$ is the distance difference between the points to be matched that are selected from the *i*-th group of point clouds to be registered, after transformation, and the corresponding points in the target point cloud.

#### 2.2.2. ICP Fine Registration

(1) Rigid body transformation matrix and its solution

The essence of point cloud registration is to ensure that the point clouds of the same object, which are measured at different times, positions and directions and in different coordinate systems, are located in the same coordinate system through rigid body transformation [27]. Rigid body transformation includes translation transformation and rotation transformation. Suppose that the two point clouds that need to be registered are the point cloud P to be registered and the target point cloud Q. Then, the transformation matrix *H* from the point cloud P to be registered to the target point cloud Q is as follows:

(6) $$H=\left[\begin{array}{cccc} \alpha_{11} & \alpha_{12} & \alpha_{13} & t_{x}\\ \alpha_{21} & \alpha_{22} & \alpha_{23} & t_{y}\\ \alpha_{31} & \alpha_{32} & \alpha_{33} & t_{z}\\ v_{x} & v_{y} & v_{z} & s \end{array}\right]$$

After simplification, it is shown as follows:

(7) $$H=\left[\begin{array}{cc} R & T\\ V & S \end{array}\right]$$

where $R=\left[\begin{array}{ccc} \alpha_{11} & \alpha_{12} & \alpha_{13}\\ \alpha_{21} & \alpha_{22} & \alpha_{23}\\ \alpha_{31} & \alpha_{32} & \alpha_{33} \end{array}\right]$ denotes the rotation matrix; $T={\left[\begin{array}{ccc} t_{x} & t_{y} & t_{z} \end{array}\right]}^{T}$ denotes the translation matrix; $V=\left[\begin{array}{ccc} v_{x} & v_{y} & v_{z} \end{array}\right]$ denotes the perspective transformation matrix; and *S* denotes the overall scale factor.

In general, the point cloud data obtained by scanning the workpiece with a 3D scanner will not be deformed. Therefore, the rigid body transformation matrix *H* is usually as shown in Equation (8):

(8) $$H=\left[\begin{array}{cc} R_{3\times 3} & T_{3\times 1}\\ V_{1\times 3} & S \end{array}\right]$$

The rotation matrix *R* and the translation matrix *T* are as shown in Equations (9) and (10), respectively:

(9) $$R_{3\times 3}=\left[\begin{array}{ccc} 1 & 0 & 0\\ 0 & \cos\alpha & \sin\alpha \\ 0 & -\sin\alpha & \cos\alpha \end{array}\right]\left[\begin{array}{ccc} \cos\beta & 0 & -\sin\beta \\ 0 & 1 & 0\\ \sin\beta & 0 & \cos\beta \end{array}\right]\left[\begin{array}{ccc} \cos\gamma & \sin\gamma & 0\\ -\sin\gamma & \cos\gamma & 0\\ 0 & 0 & 1 \end{array}\right]$$

(10) $$T_{3\times 1}={\left[\begin{array}{ccc} t_{x} & t_{y} & t_{z} \end{array}\right]}^{T}$$

where α,β,γ denote the angle of rotation of the point cloud along the x-axis, y-axis and z-axis; $t_{x},t_{y},t_{z}$ denote the translation distance of the point cloud along the x-axis, y-axis and z-axis.

From Equation (9), the rotation matrix *R*_3×3_ can be expressed via Formula (11):

(11) $$R_{3\times 3}=\left[\begin{array}{ccc} \cos\beta \cos\gamma & \cos\beta \sin\gamma & -\sin\beta \\ -\cos a\sin\gamma -\sin a\sin\beta \cos\gamma & \cos a\cos\gamma +\sin a\sin\beta \sin\gamma & \sin a\cos\beta \\ \sin a\sin\gamma +\cos a\sin\beta \cos\gamma & -\sin a\cos\gamma -\cos a\sin\beta \sin\gamma & \cos a\cos\beta \end{array}\right]$$

In order to determine the six parameters $\alpha ,\beta ,\gamma ,t_{x},t_{y},t_{z}$ in the rigid body transformation matrix described above, at least six equations are theoretically required. This requires selecting three pairs of non-collinear matching point pairs (*P*_1_, *Q*_1_), (*P*_2_, *Q*_2_) and (*P*_3_, *Q*_3_) in the overlapping area of the roughly registered point cloud data, so as to correctly calculate the parameter values of the rigid body transformation matrix, as shown in Equation (12):

(12) Q=RP+T

Let the coordinates of the point cloud data P be ${\left[\begin{array}{ccc} X_{p} & Y_{p} & Z_{p} \end{array}\right]}^{T}$ and the coordinates of Q be ${\left[\begin{array}{ccc} X_{Q} & Y_{Q} & Z_{Q} \end{array}\right]}^{T}$.

Substitute Equations (9) and (10) into Equation (12), and the result is as follows:

(13) $$\left[\begin{array}{c} X_{Q}\\ Y_{Q}\\ Z_{Q} \end{array}\right]=\left[\begin{array}{ccc} 1 & 0 & 0\\ 0 & \cos\alpha & \sin\alpha \\ 0 & -\sin\alpha & \cos\alpha \end{array}\right]\left[\begin{array}{ccc} \cos\beta & 0 & -\sin\beta \\ 0 & 1 & 0\\ \sin\beta & 0 & \cos\beta \end{array}\right]\left[\begin{array}{ccc} \cos\gamma & \sin\gamma & 0\\ -\sin\gamma & \cos\gamma & 0\\ 0 & 0 & 1 \end{array}\right]\left[\begin{array}{c} X_{P}\\ Y_{P}\\ Z_{P} \end{array}\right]+\left[\begin{array}{c} t_{x}\\ t_{y}\\ t_{z} \end{array}\right]$$

Point cloud registration aims to solve the rigid body transformation matrix between the point cloud P to be registered and the target point cloud Q. The solution process is as follows.

A quaternion is a four-dimensional vector composed of one real number and three imaginary numbers. The representation form of a quaternion is shown in Equation (14):

(14) $$a=a_{0}+a_{1}i+a_{2}j+a_{3}k={\left[\begin{array}{cccc} a_{0} & a_{1} & a_{2} & a_{3} \end{array}\right]}^{T}$$

where $a_{0},a_{1},a_{2},a_{3}$ are real numbers; i,j,k denote the generalized imaginary units. These units are perpendicular to each other and satisfy $i^{2}=j^{2}=k^{2}=-1$.

① Calculate the centroids *C_p_* and *C_q_* of point clouds P and Q, as shown in Equations (15) and (16):

(15) $$C_{p}=\frac{1}{n}\sum_{i=1}^{n}P_{i}$$

(16) $$C_{q}=\frac{1}{n}\sum_{i=1}^{n}Q_{i}$$

where n denotes the number of point clouds in point cloud data P and Q; *P_i_*, *Q_i_* denote the corresponding point pairs in the two sets of point cloud data.

② Calculate the covariance matrix, as shown in Equation (17):

(17) $$E=\frac{1}{n}\sum_{i=1}^{n}\left[(P_{i}-C_{p}){\left(Q_{i}-C_{q}\right)}^{T}\right]=\frac{1}{n}\sum_{i=1}^{n}\left[P_{i}Q_{i}^{T}\right]-C_{p}C_{q}^{T}$$

③ Solve the cyclic column vector $\Delta =\left[\begin{array}{ccc} e_{23} & e_{31} & e_{12} \end{array}\right]$ of the covariance matrix, and the three values in the formula $e_{23},e_{31},e_{12}$ can be calculated according to $e_{ij}=(E-E^{T}),(i,j=1,2,3)$.

④ Use the covariance matrix E and the cyclic column vector △ to construct a 4 × 4 symmetric matrix, as shown in Equation (18):

(18) $$M=\left[\begin{array}{cc} tr(E) & \Delta^{T}\\ \Delta & E+E^{T}-tr(E)I_{3} \end{array}\right]$$

where *I*_3_ denotes a 3 × 3 identity matrix; tr(*E*) denotes the trace of the covariance matrix *E*.

⑤ In order to determine the eigenvalue of the symmetric matrix *M* and its corresponding eigenvector, it is necessary to select the eigenvector corresponding to the maximum eigenvalue, which is the required quaternion value $a={\left[\begin{array}{cccc} a_{0} & a_{1} & a_{2} & a_{3} \end{array}\right]}^{T}$.

⑥ Solve for the rotation matrix *R* and the translation matrix *T*.

$E=U\Lambda V^{T}$ can be obtained through the SVD decomposition of the covariance matrix *E*, where Λ is the non-negative diagonal matrix composed of the eigenvalues of E. Both *U* and *V* are 3 × 3 identity orthogonal matrices. The rotation matrix *R* and the transfer matrix *T* can be calculated via Equations (19) and (20):

(19) R=a0+2a1−2a2−2a322(a1a2−a0a3)2(a1a3+a0a2)2(a1a2+a0a3)a0+2a2−2a1−2a322(a2a3−a0a1)2(a1a3−a0a2)2(a2a3+a0a1)a0+2a3−2a1−2a22

(20) $$T=C_{p}-RC_{q}$$

(2) ICP algorithm

Point cloud registration involves two known point clouds: the point cloud P to be registered and the target point cloud Q. Points $p_{i}$ are selected from the point cloud P to be registered; then, in the target point cloud Q, the points $q_{i}$ with the closest Euclidean distance are searched for. These points are paired with the selected points $p_{i}$ from P to form corresponding point pairs. The parameters of the rigid body transformation matrix are solved through continuous iteration. The parameters of the rigid body transformation matrix that minimize Equation (21) are taken as the condition for the termination of the iteration, and this is used to achieve the updating of the point cloud positions [28].

(21) $$f\left(R,T\right)=\frac{1}{k}\sum_{i=1}^{k}q_{i}-{\left(Rp_{i}+T\right)}^{2}=\min$$

where *R* is the rotation matrix; *T* is the translation matrix; and *k* is the number of corresponding points.

The ICP algorithm, through an iterative approach based on the closest point matching between point cloud data, gradually optimizes the rigid body transformation matrix. It searches for the corresponding points in the target point cloud that have the minimum distance from the source point cloud. Subsequently, by iterating this process, it gradually reduces the spatial position deviation between the two point clouds in the same coordinate system. The algorithm continues until the termination conditions of the iteration are met, thus achieving the precise alignment of the point clouds.

**Figure 5:**
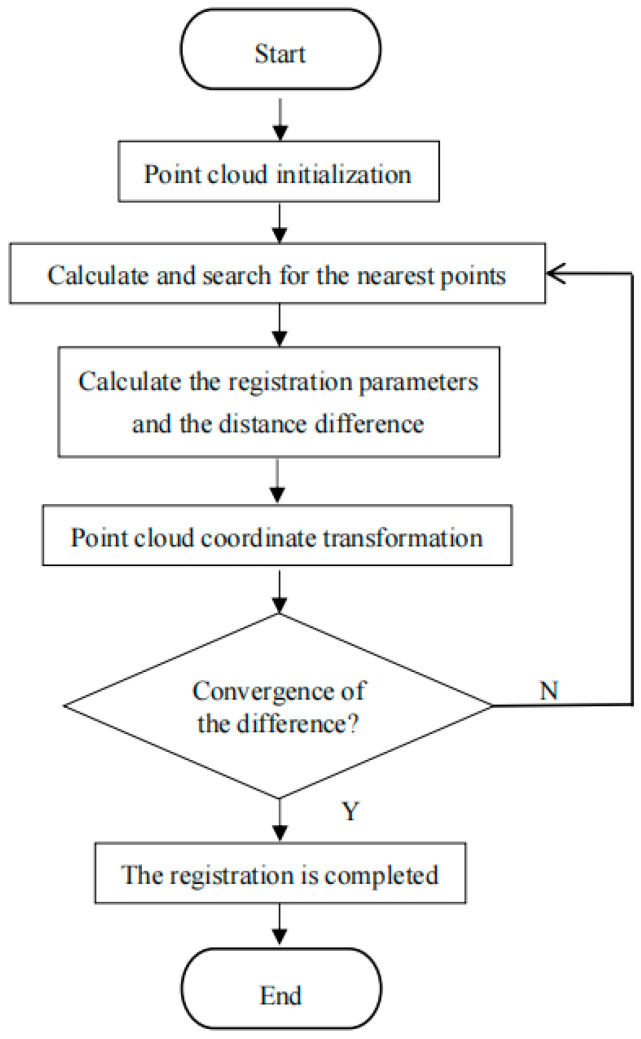
The flow chart of the ICP algorithm.

The registration process of the ICP algorithm is shown in Figure 5.

(3) Improved ICP algorithm

The ICP algorithm has relatively strict requirements for the initial position of the point cloud. By searching for the corresponding points of the point cloud to be registered in the target point cloud, since every point in the target point cloud participates in the search, the computational time is increased. During the process of searching for corresponding point pairs, the ICP algorithm may result in a large number of incorrect point pairs, which reduces the registration accuracy, affects the final registration result and makes it difficult to quickly converge to the optimal solution. More iterations are required to obtain a satisfactory registration result.

In response to the above problems existing in the ICP algorithm, this paper proposes an improved ICP algorithm as follows.

① Addressing the characteristics of point cloud data, the KD tree algorithm is adopted to realize the search for corresponding point pairs, which improves the search efficiency.

② The ISS algorithm is used to extract key feature points, and point cloud registration is carried out by matching these significant feature points, so as to improve the registration speed.

③ By combining the FPFH and SAC-IA algorithms, rough registration is achieved before the fine registration of point clouds, providing a better initial position for the point clouds. The FPFH values are calculated to express the local geometric features of the point clouds, providing more informative feature descriptors for the SAC-IA rough registration, which helps to estimate the initial corresponding relationships more accurately. The rough registration by the SAC-IA algorithm provides a better initial position for the ICP algorithm, reducing the sensitivity of the ICP algorithm to the initial position and better solving the problem whereby the ICP algorithm is prone to falling into locally optimal solutions. This helps the ICP algorithm to converge to the optimal solution more quickly, reduces the number of algorithm iterations and improves the registration efficiency.

**Figure 6:**
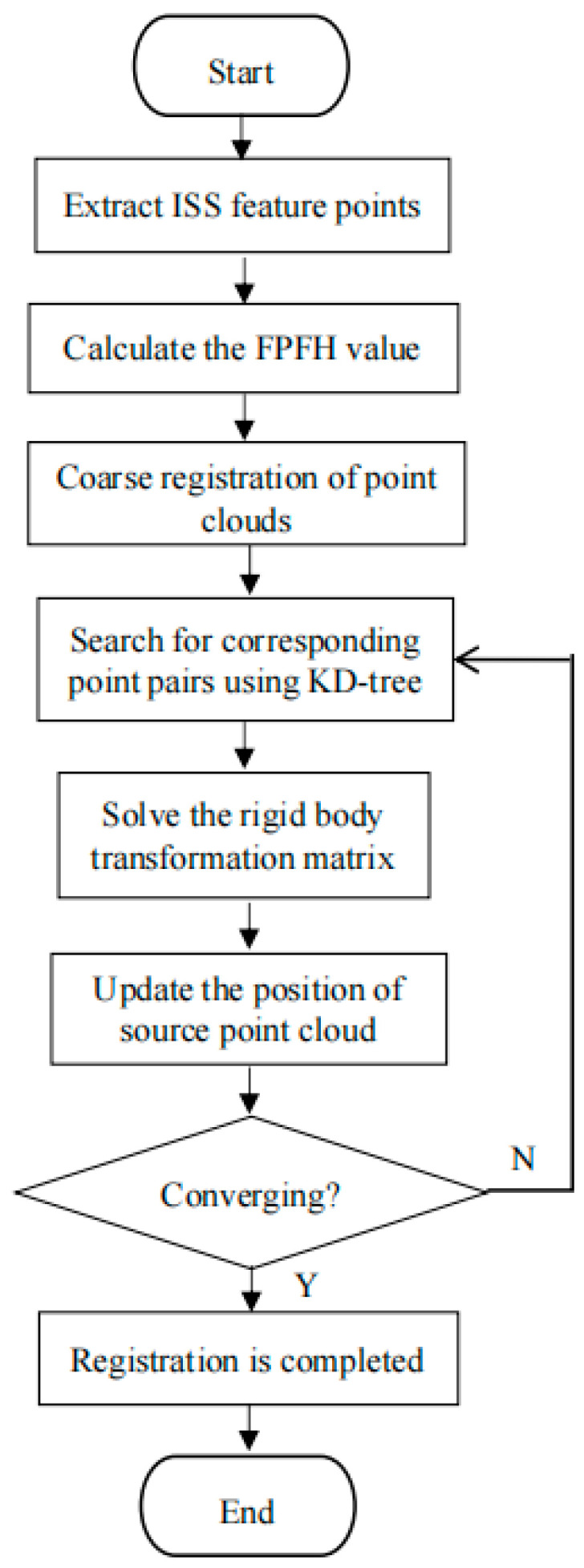
The improved ICP algorithm.

The specific steps of the improved ICP algorithm are shown in Figure 6.

### 2.3. A 3D Reconstruction Algorithm for Sheet Metal Parts

#### 2.3.1. Greedy Projection Triangulation Processing

The greedy projection triangulation algorithm is mainly used for the 3D reconstruction of point cloud data. It uses locally optimal solutions to approximately achieve the overall optimal surface structure [29]. The specific process is as follows.

**Figure 7:**
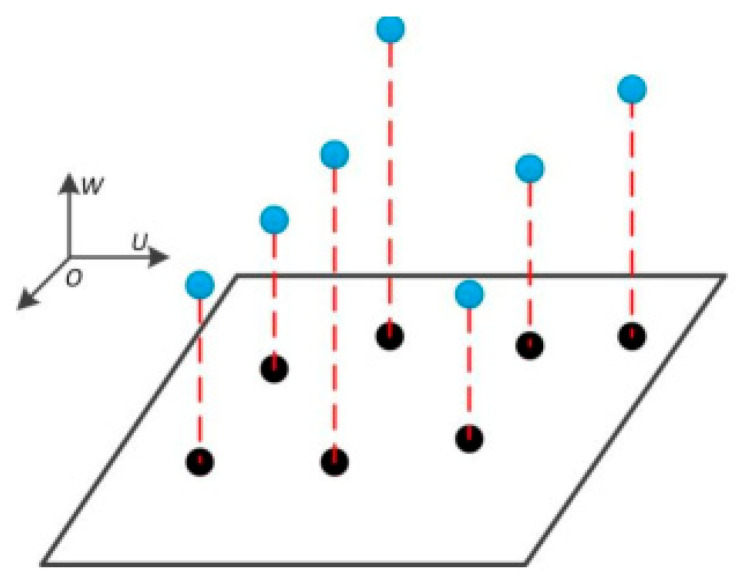
Local plane fitting and point cloud projection.

(1) Nearest neighbor search. For any point P in the point cloud data, the KD tree neighborhood search method is adopted to determine its neighborhood containing *k* nearest neighbor points.

(2) Two-dimensional projection of the local plane. Determine the projection tangent plane of a certain point P and all the points within its k-neighborhood and project all the points within the neighborhood onto this two-dimensional plane, as shown in Figure 7.

(3) Construct a spatial network. Use the greedy projection triangulation algorithm to perform planar triangulation on the points on the projection plane to determine the topological relationship. This algorithm randomly selects a point from the point cloud data as the initial growing point. Each time, a locally optimal point is selected as the new growing point. Then, according to the projection relationship, the growing points on the two-dimensional plane are mapped back to the three-dimensional space, thereby constructing the topological relationship in the three-dimensional space.

#### 2.3.2. Poisson Surface Reconstruction Processing

**Figure 8:**
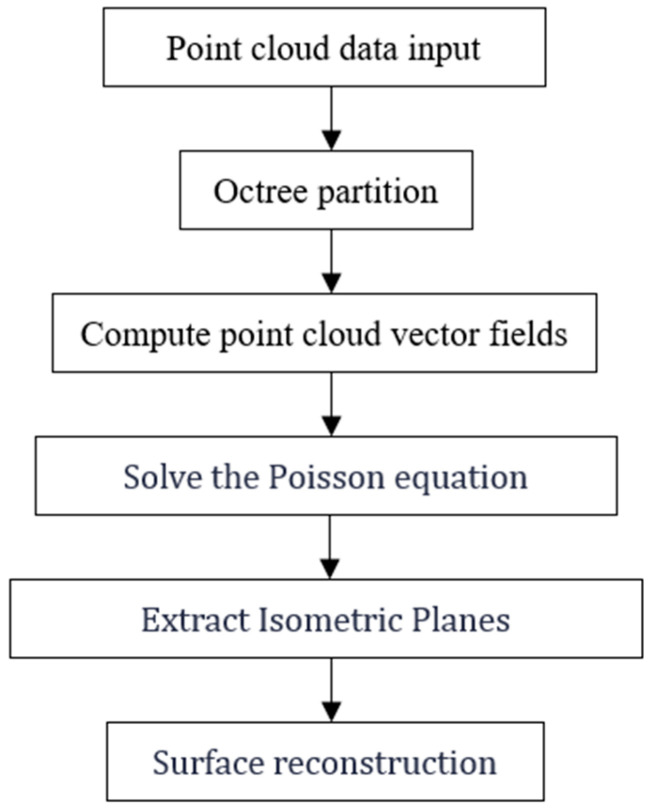
Steps of Poisson reconstruction algorithm.

Poisson reconstruction is the process of transforming 3D point cloud data into an implicit function representation. This method constructs a smooth surface model from the point cloud data by solving partial differential equations [30]. In particular, it constructs a densified function field that can represent the topological structure and geometric information of the point cloud data set P. Using the method of implicit fitting, by solving the discrete Poisson equation with the point cloud data as the boundary condition, the values of the surface function are solved. Then, the isosurface of the function field is extracted to construct a model surface that contains the complete information of the set entity, more accurately reflecting the surface features of the set and its subtle differences. The Poisson reconstruction algorithm is shown in Figure 8.

**Figure 9:**
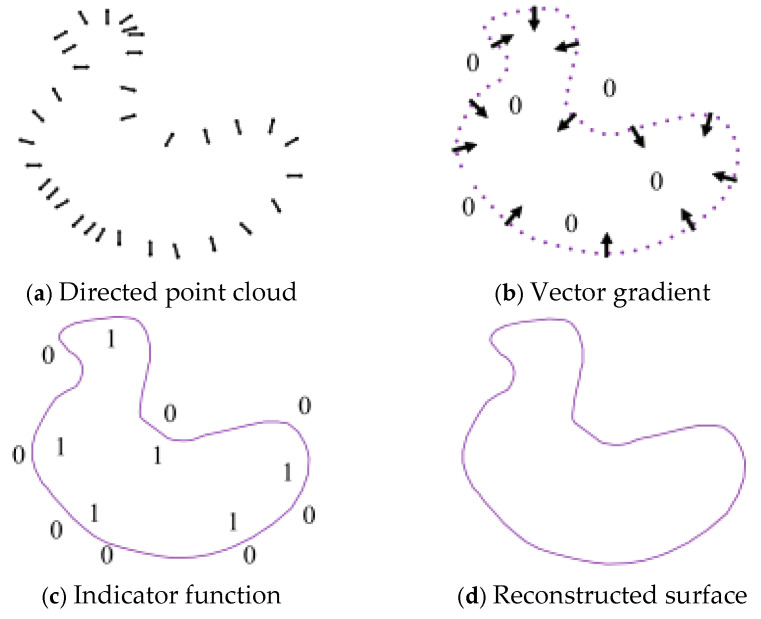
Schematic diagram of Poisson reconstruction.

If the implicit function f(x,y,z) is used to represent the surface model, the position of the point cloud data can be determined by comparing the value of the function f(x,y,z) with 0. The solution set of f(x,y,z) = 0 is the boundary of the model surface, as shown in Figure 9.

The advantage of this algorithm lies in its ability to ensure the sealing of the model reconstruction surface while accurately reflecting the geometric characteristics and subtle features of the model surface. The Poisson algorithm mainly transforms the problem of surface reconstruction into solving the Poisson equation—that is, determining the indicator function. First, an octree is constructed. Using the characteristics of the octree, the implicit function is represented. Then, the function space is set, and the vector field is created to complete the solution of the Poisson equation—that is, to solve the indicator function. Finally, the isosurface is extracted. Isosurface extraction means obtaining the surface corresponding to the sampling points on the surface of the measured object model.

#### 2.3.3. MLS Smoothing Processing

Since the scanning light cannot completely cover the surface of a smooth object, irregular point cloud data are generated, resulting in an uneven surface and even the appearance of holes, which affects the integrity of the surface information of the parts. In order to achieve a better reconstruction effect, it is necessary to smooth the point cloud data before performing 3D reconstruction. This paper uses the moving least squares (MLS) method to smooth and resample the point cloud data. The missing parts of the surface are filled through high-order polynomial interpolation, and the influence of the overlapping of the point cloud data surface after registration is eliminated.

The MLS method first selects a certain number of neighboring points around each point p in the point cloud data and establishes the relationship between the coordinates of these neighboring points and the fitting surface g as a system of constraint equations. Then, by solving the system of constraint equations, the optimal fitting surface at point p is obtained, and the point p in the original point cloud data is replaced by the points on this surface. Subsequently, the above steps are repeated continuously until the entire point cloud data are processed, so as to achieve the smoothing of the surface. It is necessary to determine the fitting function and the weight function, and the specific process is as follows.

(1)Determine the fitting function

Regard the point cloud data as a fitting data region. On a local sub-region U of this region, the fitting function *f*(x) is as shown in Equation (22):

(22) $$f(x)=\sum_{i=1}^{m}a_{i}(x)p_{i}(x)=p^{T}(x)a(x)f(x)$$

where $\alpha (x)={\left[\alpha_{1}(x),\alpha_{2}(x),\alpha_{m}(x)\right]}^{T}$ denotes the coefficients to be determined, which are functions of *x*; *m* is the number of terms in the basis function; $p(x)={\left[p_{1}(x),p_{2}(x),p_{m}(x)\right]}^{T}$ is the basis function, and it is a complete polynomial of order *k*.

For a one-dimensional problem, the basis function is $p(x)={\left[1,x,x^{2},\ldots ,x^{m}\right]}^{T}$. For a two-dimensional problem, the basis function usually has two forms: the linear basis is $p(x)={\left[1,x,y\right]}^{T},m=6;$ the quadratic basis is $p(x)={\left[1,u,v,u^{2},uv,v^{2}\right]}^{T},m=6$. In practical situations, the quadratic basis is more frequently used. To summarize, the fitting function f(x) can be expressed as in Equation (23):

(23) $$f(x)=a_{0}(x)+a_{1}(x)u+a_{2}(x)v+a_{3}(x)u^{2}+a_{4}(x)v^{2}+a_{5}(x)uv$$

In order to obtain more accurate local fitting, it is necessary to minimize the weighted sum of the squares of the local fitting differences between $f(x_{i})$ and the nodal values $y_{i}$. Therefore, the discrete weighted norm can be described as

(24) $$\begin{array}{l} J=\sum_{i=1}^{n}w(x-w_{i})[f(x)-y_{i}]\\ =\sum_{i=1}^{n}w(x-x_{i}){\left[p^{T}(x)a(x)-y_{i}\right]}^{2}\\ =\sum_{i=1}^{n}w(x-x_{i})[(a_{0}(x)+a_{1}(x)u+a_{2}(x)v+\\ +a_{3}(x)u^{2}+a_{4}(x)v^{2}+a_{5}(x)uv)-y_{i}{]}^{2} \end{array}$$

where n is the number of nodes in the influence area; f(x) is the fitting function; $y_{i}=y(x_{i})$ is the nodal value at the node $x=x_{i}$; and $w(x-x_{i})$ is the weight function at the node $x=x_{i}$.

In order to determine the coefficient a(x), Equation (24) should take a minimum value. By differentiating with respect to a(x) and setting $\frac{\partial J}{\partial q}=0$, the equation can be obtained as follows:

(25) $$a={\left(BWB\right)}^{-1}BWy$$

where $B=\left[\begin{array}{cccccc} 1 & u_{1} & v_{1} & u_{1}^{2} & u_{1}v_{1} & v_{1}^{2}\\ \vdots & \vdots & \vdots & \vdots & \vdots & \vdots \\ 1 & u_{n} & v_{n} & u_{n}^{2} & u_{n}v_{n} & u_{n}^{2} \end{array}\right]$;

W is an n-order diagonal matrix, and $W=\left[\begin{array}{cccc} w(x-x_{1}) & 0 & \ldots & 0\\ 0 & w(x-x_{2}) & \ldots & 0\\ \vdots & \vdots & \vdots & \vdots \\ 0 & 0 & \ldots & w(x-x_{n}) \end{array}\right]$;

$y={\left[y(x_{1}),y(x_{2}),\ldots ,y(x_{n})\right]}^{T}$.

(2) Determine the weight function

For the MLS method, the determination of the weight function is of crucial importance for the smoothing of point cloud data. Usually, a circular region is selected as the support domain of the weight function, and its radius is defined as *r*. The weight function *w*(*x* − *x_i_*) has the following characteristics.

① Compact support: The weight function *w*(*x* − *x_i_*) is non-zero within a specific sub-domain and zero outside the sub-domain.

② Non-negativity: The values of the weight function are always non-negative. Within the support domain, the values of the weight function are always greater than zero, which ensures that the weight function has a positive impact on the data points within the selected region.

③ Smoothness: The weight function *w*(*x* − *x_i_*) is a smooth and continuous function, which helps to ensure that a smooth fitting surface can be obtained during the processing of the point cloud data and improves the fitting accuracy for local data.

④ Monotonic decrease: Within the support domain, the value of the weight function *w*(*x* − *x_i_*) decreases monotonically as $\left\|x-x_{i}\right\|$ increases.

A commonly used weight function is the cubic spline weight function, and its expression is shown in Equation (26):

(26) $$w(x)=\left\{\begin{array}{c} \frac{2}{3}-4x^{2}+4x^{3},0<x⩽0.5\\ \frac{3}{4}{\left(1-x\right)}^{3},0.5<x⩽1.0\\ 0,x>1.0 \end{array}\right.$$

where $x=\frac{r_{i}}{\beta h_{i}},r_{i}=\left\|x-x_{i}\right\|$; $h_{i}$ is the size of the support domain of the weight function for the *i*-th node; *β* is the influence coefficient, and its value usually ranges between 1.25 and 2.5.

## 3. Digital Detection Experiments on Sheet Metal Parts Based on 3D Point Clouds

### 3.1. Experiments on Point Cloud Denoising of Sheet Metal Parts

**Figure 10:**
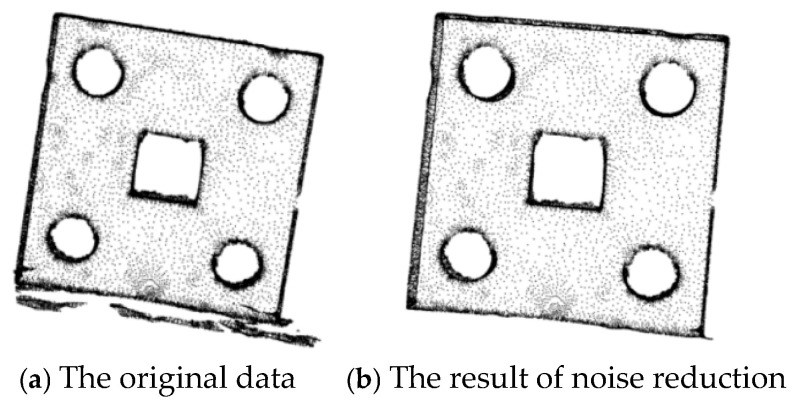
The 3D point cloud of a sheet metal part and the result of its noise reduction through pass-through filtering.

The point cloud of a sheet metal part scanned by a 3D scanner is shown in Figure 10a. In order to remove irrelevant point clouds such as the workbench and the surrounding environment, the pass-through filtering algorithm is adopted for noise reduction, as shown in Figure 10b.

**Figure 11:**
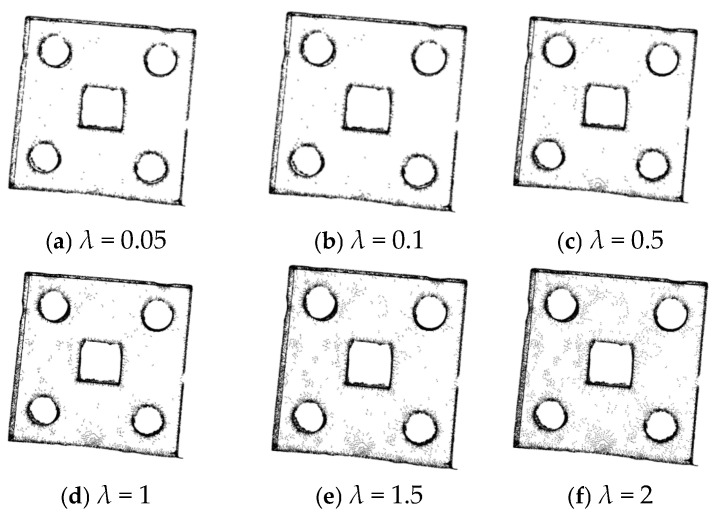
Effects of noise removal from sheet metal part through statistical filtering at different values of *λ*.

The 3D point cloud obtained after the pass-through filtering process is subjected to statistical filtering. When *k* = 1, the processing results with a change in the value of *λ* are as shown in Figure 11, and the experimental result data are presented in Table 2.

According to the filtering effect shown in Figure 11 and the number of point clouds after noise reduction in Table 2, when *k* = 1, as *λ* increases, the number of point clouds removed gradually decreases, and the number of points retained on the front-view plane of the sheet metal part gradually increases. However, the number of retained invalid points also increases accordingly, resulting in an increase in the subsequent processing time. When *λ* is less than 1, the number of point clouds removed is excessive, which may affect the effects of subsequent point cloud registration and 3D reconstruction.

**Figure 12:**
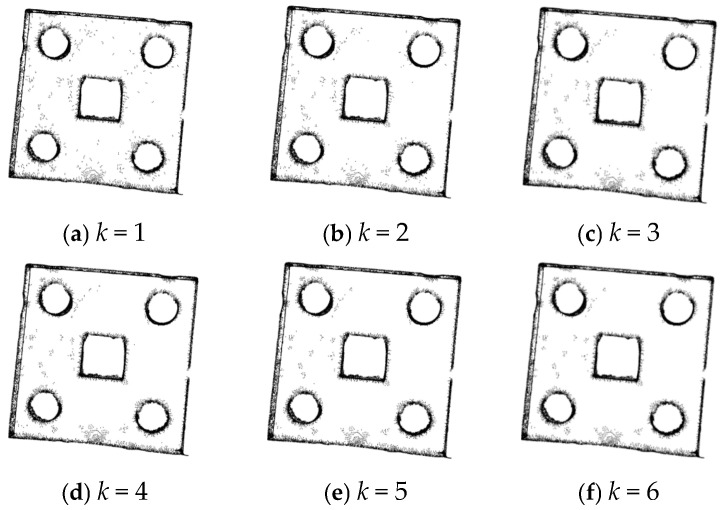
Effects of noise removal from sheet metal part through statistical filtering at different values of *k*.

When *λ* = 1, the filtering effects when changing the value of *k* are as shown in Figure 12, and the experimental data are shown in Table 3.

According to Figure 12 and Table 3, when the value of *k* changes, the number of point clouds does not change significantly. Therefore, the value of k has little impact on the number of point clouds. Based on the above analysis, *λ* = 1 and *k* = 5 are ultimately selected.

**Figure 13:**
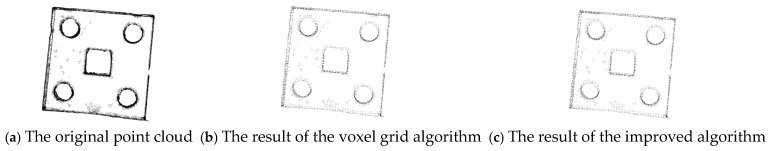
Downsampling of sheet metal parts.

Figure 13 shows a schematic diagram of the downsampling of sheet metal parts. Here, Figure 13a shows the original point cloud data, Figure 13b shows the processing results after downsampling by voxel grid filtering, and Figure 13c shows the processing results of the improved downsampling algorithm. Table 4 shows the number of point clouds after processing by different downsampling algorithms.

According to Figure 13 and Table 4, the number of point clouds after downsampling by the improved algorithm is the same as that after downsampling by the voxel grid method. However, the improved algorithm can preserve the characteristics of the point clouds well and has the optimal downsampling effect.

### 3.2. Registration Experiments on Sheet Metal Parts

**Figure 14:**
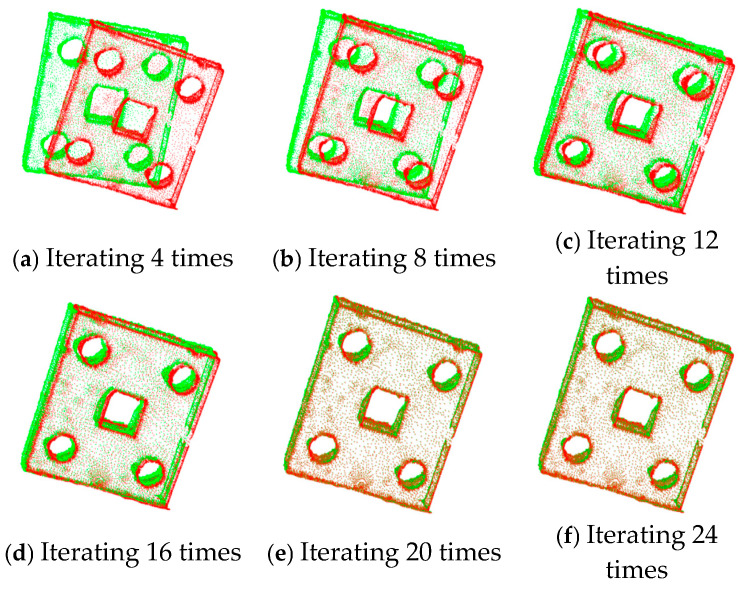
Registration of sheet metal parts using ICP algorithm.

**Figure 15:**
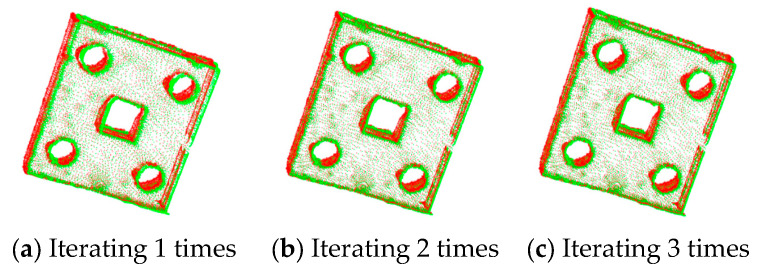

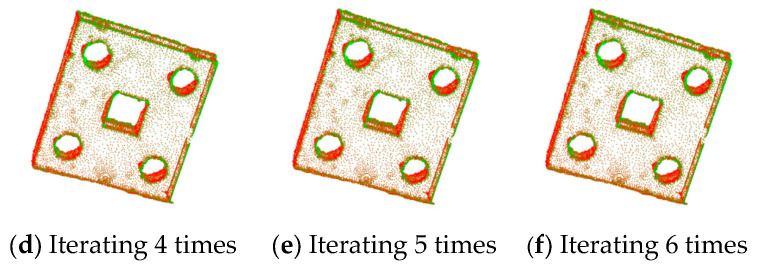
Registration of sheet metal parts using the improved algorithm.

The ICP registration algorithm and the improved algorithm are used to register the sheet metal parts. The registration results are shown in Figure 14 and Figure 15, respectively.

The statistical results in terms of the registration errors and time consumption for sheet metal parts are shown in Table 5.

From the experimental results, it can be observed that, when the ICP algorithm is applied to register sheet metal parts, if the number of iterations is low, obvious registration errors will occur. Increasing the number of iterations can gradually optimize the registration effect, but the corresponding registration time will also increase, resulting in a decrease in registration efficiency. Since the ICP algorithm is highly sensitive to small changes in the initial values, this affects its overall registration performance.

**Figure 16:**
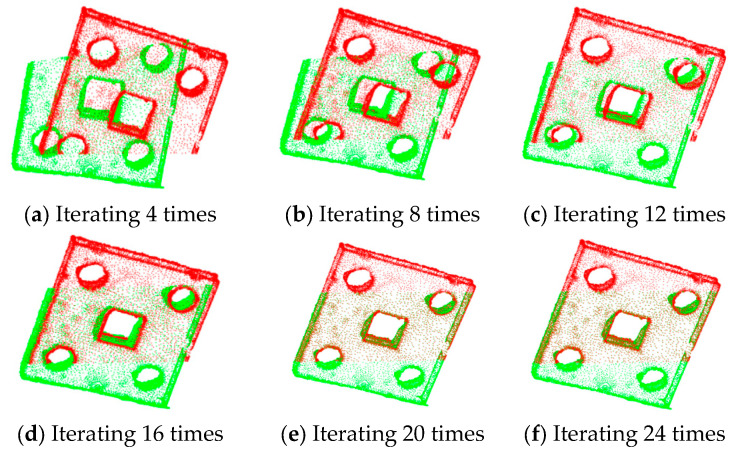
Registration of partially overlapping sheet metal parts using the ICP algorithm.

**Figure 17:**
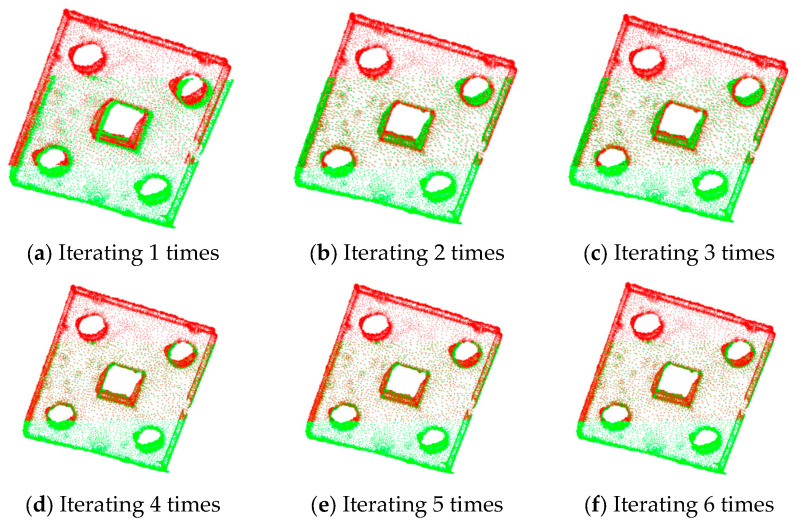
Registration of partially overlapping sheet metal parts using the improved algorithm.

The results of ICP with different numbers of iterations for partially overlapping sheet metal parts are shown in Figure 16. The results of the improved algorithm with different numbers of iterations are shown in Figure 17.

The statistical results in terms of the registration errors and time consumption for partially overlapping sheet metal parts are shown in Table 6.

According to Figure 16 and Figure 17 and Table 6, when the ICP algorithm is applied to register sheet metal parts, a small number of iterations results in the poor overlapping of the two sets of point clouds, and obvious registration errors occur. Compared with the direct application of the ICP algorithm, the improved method proposed in this paper, with an increase in the number of iterations, can not only significantly improve the registration accuracy but also effectively reduce the registration time, thus improving the registration efficiency.

### 3.3. Three-Dimensional Reconstruction Experiments on Sheet Metal Parts

**Figure 18:**
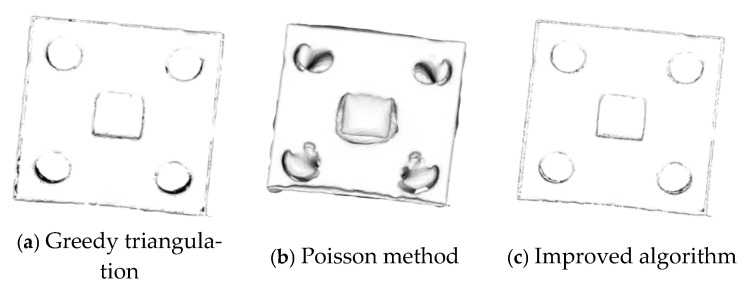
The 3D reconstruction results for sheet metal parts.

**Figure 19:**
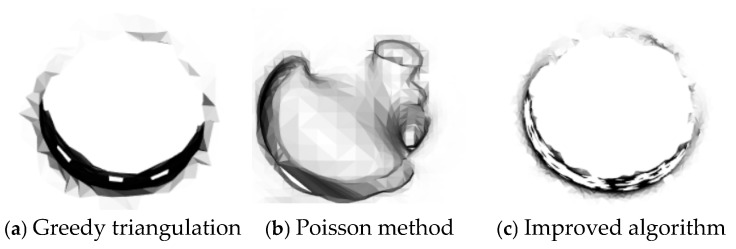
Locally enlarged views of the 3D reconstruction of sheet metal parts.

The sheet metal parts after registration by the improved algorithm are subjected to 3D reconstruction, as shown in Figure 18. The reconstructed model is magnified locally, as shown in Figure 19. In both Figure 18 and Figure 19, (a) represents the greedy triangulation reconstruction, (b) represents the Poisson reconstruction, and (c) represents the reconstruction results of the improved algorithm.

A comparison of the running times of the three different reconstruction algorithms applied to sheet metal parts is carried out, and the results are shown in Table 7.

According to Figure 18 and Figure 19 and Table 7, the surface of the sheet metal part after greedy reconstruction is not smooth and has many sharp edges and corners. The surface of the sheet metal part after Poisson reconstruction is smooth, but it performs poorly in the reconstruction of circular and square holes and cannot achieve accurate shapes. The surface model generated by the Poisson reconstruction algorithm exhibits excellent performance both in terms of overall consistency and surface smoothness. However, when dealing with objects that have multiple interconnected parts, it cannot effectively reconstruct each part individually; instead, it creates a continuous overall surface. In addition, for objects with relatively complex shapes and large variations in the density of surface point clouds, the features of the reconstructed surface model cannot be clearly expressed. The improved greedy reconstruction can not only reconstruct the shape of the sheet metal parts but also produces fewer sharp edges and corners and a smooth surface. Although the reconstruction time of the improved algorithm is longer than that of greedy reconstruction, it has a better reconstruction effect. Therefore, this paper uses the improved algorithm to complete the 3D reconstruction of sheet metal parts.

### 3.4. Digital Detection Experiments on Sheet Metal Parts

#### 3.4.1. Digital Detection Algorithm

(1) Straight-line detection algorithm

Since a point in the image space corresponds to a unique equation in the parameter space, the Hough transform can be used for straight-line detection. By utilizing the duality property between points and lines, and conducting statistical analysis on the point set in the image, the precise identification of straight lines can be achieved.

In the Cartesian coordinate system XOY, the straight line passing through point$\left(x_{0},y_{0}\right)$ is as shown in Equation (27):

(27) $$y_{0}=kx_{0}+b\;a={\left(BWB\right)}^{-1}BWy$$

where *k* is the slope of a straight line, and *b* is the intercept of a straight line.

At the same time, the above formula can be rewritten as follows:

(28) $$b=-kx_{0}+y_{0}\;a={\left(BWB\right)}^{-1}BWy$$

**Figure 20:**
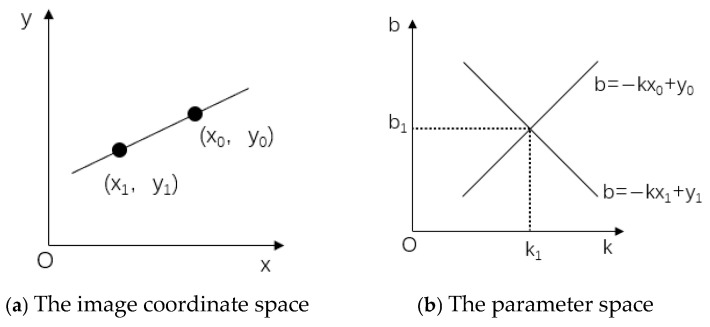
The Hough transform in the Cartesian coordinate system.

Point $\left(x_{0},y_{0}\right)$ in the image space is mapped to a straight line in the parameter space through the Hough transform, and the equation of this straight line is uniquely determined. Similarly, point $\left(x_{1},y_{1}\right)$ in the image space also corresponds to a straight line in the parameter space, and the straight line corresponding to point $\left(x_{1},y_{1}\right)$ intersects with the straight line corresponding to point $\left(x_{0},y_{0}\right)$ at point $\left(k_{1},b_{1}\right)$ in the parameter space, as shown in Figure 20.

Therefore, any straight line passing through points $\left(x_{0},y_{0}\right)$ and $\left(x_{1},y_{1}\right)$ in the image space corresponds to a straight line in the k-b parameter space. If these straight lines intersect at a point $\left(k_{1},b_{1}\right)$ in the parameter space, then $k_{1}$ and $b_{1}$ are the parameters of the straight line connecting $\left(x_{0},y_{0}\right)$ and $\left(x_{1},y_{1}\right)$ in the image space.

The shape of the curve in the parameter space is determined by the curve function in the original image space. In order to avoid the situation whereby the slope of a straight line may be infinite, a common practice is to transform the image pixels from the Cartesian coordinate system (x,y) to the polar coordinate system (r,θ). In this coordinate system, the equation of a straight line can be represented by the polar radius r and the polar angle θ, as shown in Equation (29):

(29) $$r=x\cos\theta +y\sin\theta ,r>0,0<\theta <2\pi \;a={\left(BWB\right)}^{-1}BWy$$

where r is the distance from the line to the origin of the coordinate; θ is the angle between the horizontal direction and the vertical line of the detection line.

In the cumulative analysis of the polar coordinate system, each intersecting straight line is assigned a unit weight. The weight of an intersection point is determined by the sum of the weights of all straight lines passing through that point. If multiple straight lines intersect at the same point, a higher sum of weights indicates that the straight line corresponding to this point is composed of a large number of points. By setting a specific parameter accumulator L(k,b), the accumulator $L(k_{0},b_{0})$ corresponding to the intersection point will be gradually accumulated. When the value of the accumulator reaches a peak, the parameter (r,θ) of this point represents a straight line in the Cartesian coordinate system.

(2) Circle detection algorithm

The Hough transform is widely used in the recognition of circular contours in two-dimensional image analysis. It can effectively distinguish circular structures from complex backgrounds and maintain stable performance even in the presence of noise interference. The equation of a circle in the known Cartesian coordinate system is

(30) $${\left(x-a\right)}^{2}+{\left(y-b\right)}^{2}=r^{2}$$

The transformation from the Cartesian coordinate system to the polar coordinate system can be expressed as

(31) $$\left\{\begin{array}{c} a=x-r\cos\theta \\ b=y-r\sin\theta \end{array}\right.$$

In a three-dimensional coordinate system composed of the parameters *a*, *b* and *r*, any point can represent a circle. If the center point and the radius of a circle in an image are known, then, by rotating the coordinates of any point on the circle around the center point, the polar coordinates of all points on the circle can be obtained.

#### 3.4.2. Projection Experiments on Sheet Metal Parts

This paper adopts a method that combines point cloud and image processing to improve the contour-based measurement method. The key step of this method is to flatten the 3D point cloud. The main steps are as follows:

(1) Perform 3D reconstruction on the point cloud data obtained by scanning the workpiece with a 3D scanner;

(2) Project the 3D reconstructed model onto a plane to obtain a 2D graphic.

**Figure 21:**
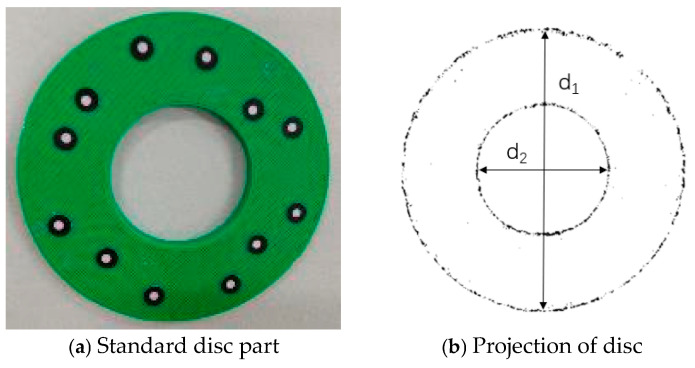
A disc and its planarized image.

Figure 21a shows a standard disc part, and an image of its projection onto a plane is shown in Figure 21b.

**Figure 22:**
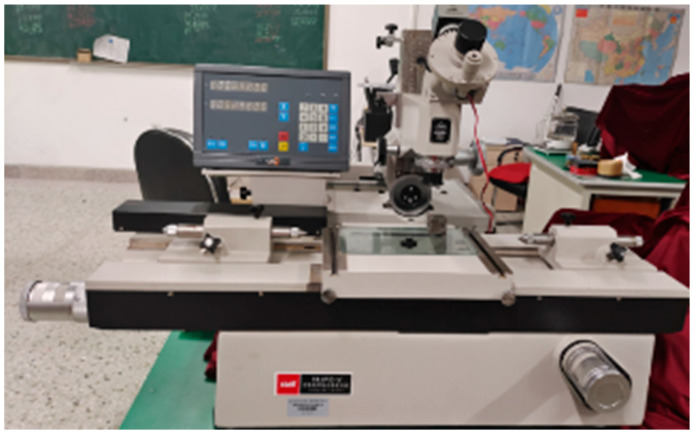
The universal tool microscope.

The dimensions of the standard disc part are obtained by measuring it with a universal tool microscope. The pixel values of the disc projection are measured by the Hough algorithm. The universal tool microscope is shown in Figure 22, and the measurement results are shown in Table 8.

From Table 8, it can be concluded that the ratio of the dimensions of the standard disc part to the pixel values is 0.0633. When the 3D scanner scans the sheet metal part at the same distance, the ratio of the measured dimensions to the pixel values remains unchanged. This value is taken as the conversion scale factor for the scanning of the sheet metal part. The measured dimensions of the sheet metal part can be obtained through this scale factor and the pixel values of the sheet metal part.

The dimensions of the sheet metal part are measured by a universal tool microscope, and these are regarded as the actual dimensions of the sheet metal part. The dimensions of the workpiece obtained by measuring the sheet metal part through the digital detection system are the measured values. The measured values are compared with the actual dimensions of the sheet metal part measured by the universal tool microscope to obtain the measurement error of the digital detection system.

#### 3.4.3. Experiments on Dimension Measurement of Sheet Metal Parts

**Figure 23:**
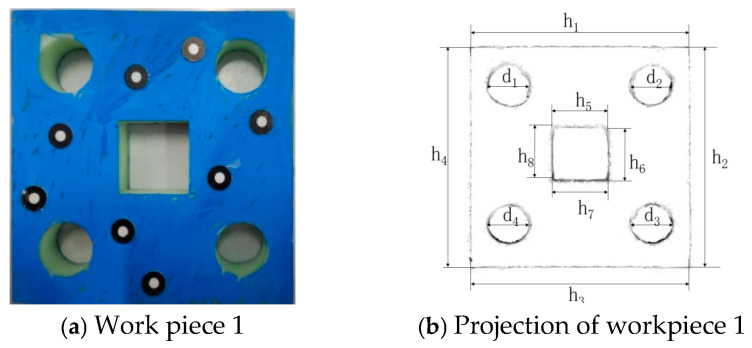
Workpiece 1 and its projection image.

Workpiece 1 is shown in Figure 23a. The image obtained by projecting the 3D model of the sheet metal part, which is acquired through the 3D reconstruction of the point cloud data scanned by a 3D scanner, onto a plane is shown in Figure 23b. The measurement results are shown in Table 9.

**Figure 24:**
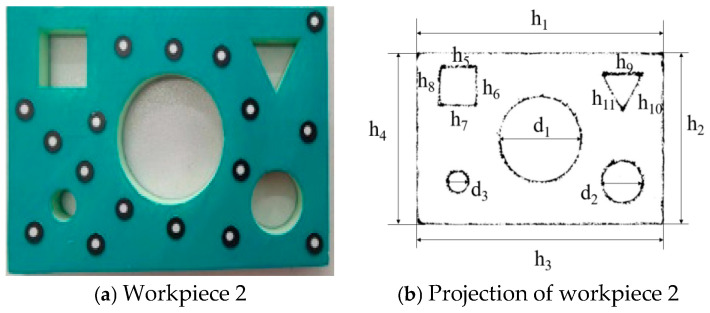
Workpiece 2 and its projection image.

According to Table 9, the minimum error value of the dimensions measured for this sheet metal part by the digital detection system is 0.1416 mm, and the maximum is 0.2637 mm, both of which are less than 0.3 mm. The measurement accuracy of the digital detection system for workpiece 1 meets the technical requirements. Workpiece 2 is shown in Figure 24a. The image obtained by projecting the 3D model of the sheet metal part, which is acquired through the 3D reconstruction of the point cloud data scanned by a 3D scanner, onto a plane is shown in Figure 24b. The measurement results are shown in Table 10.

**Figure 25:**
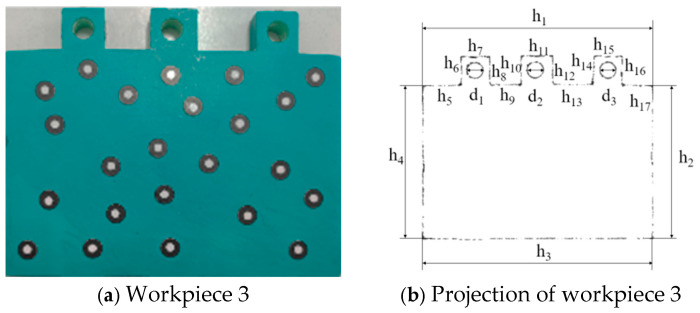
Workpiece 3 and its projection image.

According to Table 10, the minimum error value of the dimensions measured for this sheet metal part by the digital detection system is 0.1559 mm, and the maximum is 0.2672 mm, both of which are less than 0.3 mm. The measurement accuracy of the digital detection system for workpiece 2 meets the technical requirements. Workpiece 3 is shown in Figure 25a. The image obtained by projecting its 3D reconstruction onto a plane is shown in Figure 25b. The measurement results are shown in Table 11.

According to Table 11, the minimum error value of the dimensions measured for this sheet metal part by the digital detection system is 0.1443 mm, and the maximum is 0.2684 mm, both of which are less than 0.3 mm. The measurement accuracy of the digital detection system for workpiece 3 meets the technical requirements.

Based on the measurement results in Table 9, Table 10 and Table 11, the minimum error value of the digital detection system when measuring the three parts is 0.1416 mm, and the maximum error value is 0.2684 mm. The main reasons for the occurrence of errors are as follows:

(1) The surface of the model after 3D reconstruction is not smooth enough. As can be seen from Figure 18, the surface of the 3D model of the reconstructed sheet metal part has sharp edges and corners, and it is not completely consistent with the sheet metal part, resulting in an inaccurate projected image.

(2) There are errors in the 3D scanner.

(3) The parameters of the point cloud processing algorithm are not the optimal parameters.

## 4. Conclusions

This paper presents a digital inspection technology for sheet metal parts based on 3D point clouds, which integrates point cloud acquisition, denoising, registration, 3D reconstruction and dimensional measurement. Using an EinScan Pro 2X 3D scanner to acquire point cloud data, this technology establishes topological relationships via KD trees, denoises through pass-through filtering and applies an improved voxel grid downsampling algorithm that retains fine features while reducing the data volume by 80.9%. For point cloud registration, the ISS algorithm extracts feature points described by FPFH, with SAC-IA for rough registration and an improved ICP algorithm (incorporating KD tree nearest-neighbor search and feature point matching), achieving 75% fewer iterations and an 84.7% lower registration error rate compared to traditional ICP. Moreover, 3D reconstruction adopts an MLS-optimized greedy projection triangulation algorithm, which outperforms Poisson reconstruction in preserving geometric features like holes. Dimensional measurement, via 3D-to-2D projection and the Hough transform with a 0.0633 scale factor, yields measurement errors of 0.1416–0.2684 mm across three workpieces, meeting the ±0.3 mm accuracy requirement, thus providing a reliable digital inspection solution for sheet metal parts.

## Figures and Tables

**Table 1:** Parameters of 3D scanner.

Device Model	EinScan Pro 2X
Scanning precision	0.045 mm
Scanning range per single scan	150 × 120 mm~250 × 200 mm
Working center distance	400 mm
Printable data output format	OBJ, STL, ASC, PLY, P3, 3MF
Weight of scanning head	1.13 kg
System requirements	Windows 10, 64-bit

**Table 2:** Test results after removing the noise of sheet metal parts with different proportional coefficients.

Serial Number	Number of Point Clouds	*λ*	*k*	Number of Point Clouds After Noise Reduction
(a)	20,463	0.05	1	13,713
(b)	20,463	0.1	1	14,120
(c)	20,463	0.5	1	16,552
(d)	20,463	1	1	17,901
(e)	20,463	1.5	1	18,674
(f)	20,463	2	1	19,253

**Table 3:** Test results after removing the noise of sheet metal parts at different adjacent points.

Serial Number	Number of Point Clouds	*λ*	*k*	Number of Point Clouds After Noise Reduction
(a)	17,901	1	1	17,901
(b)	17,901	1	2	17,761
(c)	17,901	1	3	17,687
(d)	17,901	1	4	17,622
(e)	17,901	1	5	17,617
(f)	17,901	1	6	17,616

**Table 4:** Number of point clouds for downsampling of sheet metal parts.

Algorithm	Number of Point Clouds
Initial point clouds	17,617
Downsampling by the voxel grid algorithm	3359
Downsampling by the improved algorithm	3359

**Table 5:** The registration results for sheet metal parts.

Algorithm	Number of Iterations	Registration Error (m)	Time (s)
ICP algorithm	4	8.00264 × 10^−5^	5.936
	8	1.93961 × 10^−5^	8.453
	12	5.56931 × 10^−6^	10.867
	16	1.23578 × 10^−6^	13.349
	20	6.96264 × 10^−7^	15.896
	24	2.63578 × 10^−7^	18.345
Improved algorithm	1	2.46069 × 10^−6^	11.522
	2	2.03659 × 10^−6^	11.572
	3	1.60361 × 10^−6^	11.634
	4	1.18995 × 10^−6^	11.681
	5	7.51249 × 10^−7^	11.736
	6	4.22656 × 10^−7^	11.793

**Table 6:** The registration results for partially overlapping sheet metal parts.

Algorithm	Number of Iterations	Registration Error (m)	Time (s)
ICP algorithm	4	7.07786 × 10^−5^	5.032
	8	1.89264 × 10^−5^	7.982
	12	6.68919 × 10^−6^	10.975
	16	1.52197 × 10^−6^	14.063
	20	5.69119 × 10^−7^	17.192
	24	2.03491 × 10^−7^	20.038
Improved algorithm	1	2.59116 × 10^−6^	10.581
	2	2.10359 × 10^−6^	10.623
	3	1.68134 × 10^−6^	10.674
	4	1.20319 × 10^−6^	10.716
	5	8.76916 × 10^−7^	10.759
	6	4.95649 × 10^−7^	10.807

**Table 7:** Time consumed by different algorithms for 3D reconstruction.

Algorithm	Time (s)
Greedy triangulation algorithm	4.368
Poisson algorithm	4.136
Improved algorithm	4.697

**Table 8:** Measurement results regarding the actual dimensions of the disc and its pixel values.

Size Name	Value Measured by the Universal Tool Microscope (mm)	Pixel Value
d1	95.9941	1517
d2	43.9887	695

**Table 9:** Dimensional measurement results for workpiece 1.

Dimension Annotation	Value Measured by the Universal Tool Microscope (mm)	Measured Value (mm)	Error (mm)
h1	80.0129	80.2011	0.1882
h2	80.0067	80.2644	0.2577
h3	80.0117	80.2011	0.1894
h4	79.9962	80.1378	0.1416
d1	15.0165	14.8122	0.2043
d2	15.0097	15.1922	0.1825
d3	15.0126	14.7489	0.2637
d4	15.0203	15.2553	0.2350
h5	20.0105	20.2562	0.2457
h6	20.0107	19.7496	0.2611
h7	20.0096	20.1927	0.1831
h8	20.0148	20.1927	0.1779

**Table 10:** Dimensional measurement results for workpiece 2.

Dimension Annotation	Value Measured by the Universal Tool Microscope (mm)	Measured Value (mm)	Error (mm)
h1	120.0026	120.2067	0.2041
h2	80.0103	80.2011	0.1908
h3	120.0067	120.2704	0.2637
h4	79.9972	80.2644	0.2672
h5	18.0065	18.2304	0.2239
h6	18.0112	18.1671	0.1559
h7	18.0130	17.8506	0.1624
h8	17.9826	18.1671	0.1845
h9	20.0192	20.2560	0.2368
h10	19.9763	20.1927	0.2164
h11	20.0125	20.1927	0.1802
d1	40.0070	40.2588	0.2518
d2	20.0108	19.7496	0.2612
d3	10.0203	10.2546	0.2343

**Table 11:** Dimensional measurement results for workpiece 3.

Dimension Annotation	Value Measured by the Universal Tool Microscope (mm)	Measured Value (mm)	Error (mm)
h1	120.0116	120.2067	0.1951
h2	80.0126	80.2011	0.1885
h3	119.9776	120.1434	0.1658
h4	79.9863	80.1378	0.1515
h5	20.0067	20.2561	0.2494
h6	15.0026	15.2553	0.2527
h7	15.0120	15.1924	0.1804
h8	14.9773	15.1287	0.1514
h9	17.9926	17.7242	0.2684
h10	15.0028	14.8122	0.1906
h11	15.0102	15.2553	0.2451
h12	15.0113	14.8126	0.1987
h13	21.0036	20.8257	0.1779
h14	15.0096	15.2553	0.2457
h15	15.0198	14.8755	0.1443
h16	15.0114	14.8122	0.1992
h17	15.9727	16.2048	0.2321
d1	8.0076	7.8492	0.1584
d2	8.0103	8.2290	0.2187
d3	8.0060	8.1657	0.1597

## Data Availability

The data supporting the findings of this study are available from the corresponding author upon reasonable request.
